# Fukuchi-Manabe Score for Infection Control Measures During the Very Early COVID-19 Pandemic Period When Access to Reverse Transcription-Polymerase Chain Reaction Testing Was Poor in Japan: A Single-Center Observational Prospective Cohort Study

**DOI:** 10.7759/cureus.54748

**Published:** 2024-02-23

**Authors:** Takahiko Fukuchi, Noriko Oyama-Manabe, Hitoshi Sugawara

**Affiliations:** 1 Department of Comprehensive Medicine, Division of General Medicine, Jichi Medical University, Saitama Medical Center, Saitama, JPN; 2 Department of Radiology, Jichi Medical University, Saitama Medical Center, Saitama, JPN

**Keywords:** infection control measures, quarantine, risk-scoring strategy, sars-cov-2, covid-19

## Abstract

Background: During the early stages of the COVID-19 pandemic in Japan, access to reverse transcription-polymerase chain reaction (RT-PCR) testing was limited. All patients with COVID-19 required hospitalization, and isolation of suspected COVID-19 patients had not yet been implemented. With the recently acquired evidence on COVID-19, it is important to develop a risk stratification system for isolation rooms in the context of limited resources for better resource management.

Objective: This study aimed to develop and validate a COVID-19 risk-scoring strategy, the Fukuchi-Manabe score, to safely stratify and manage isolation rooms, personal protective equipment (PPE), and RT-PCR testing in the context of limited RT-PCR testing and a short supply of PPE.

Methods: This single-center prospective study consecutively enrolled suspected COVID-19 adult inpatients between March 1 and August 31, 2020. The primary and secondary outcomes were a positive RT-PCR test and the occurrence of nosocomial infections during the study period, respectively. Factors related to patient history, symptoms, chest computed tomography findings, and laboratory data suggestive of COVID-19 were scored, totaled, and divided into four categories ("probable," "possible," "less likely," and "non-suspicious") based on the likelihood of COVID-19. Sensitivity, specificity, and positive and negative predictive values were evaluated for each probability category.

Findings: Twenty of 224 inpatients were positive on the RT-PCR test, including 18 "probable" patients (90.0%), one "possible" patient, and one "less likely" patient. The area under the curve (AUC) (95% confidence interval: 0.841-0.977), sensitivity, and specificity were 0.909, 90.0%, and 80.4%, respectively. The positive and negative predictive values and accuracy for the "probable" category were 0.90, 0.80, and 0.82, respectively. The mean and standard deviation of AUCs, validated by bootstrap analysis, were 0.910±0.034. No nosocomial infections were observed.

Conclusion: The Fukuchi-Manabe score will be helpful when other novel pathogens emerge in the future before the availability of genetic testing methods.

## Introduction

COVID-19, caused by SARS-CoV-2, is an infectious disease that continues to threaten public health and medical systems worldwide. At least 100 million cases and 3 million deaths were confirmed in the first half of 2020 [1,2]. Although there has been extensive research regarding the pathogenesis, epidemiology, management, prevention, complications, and treatment of COVID-19 [3,4], there is little evidence regarding the diagnosis and quarantine of possible patients with COVID-19 [5,6]. This was particularly relevant in the early pandemic stage when access to reverse transcription-polymerase chain reaction (RT-PCR) testing was limited.

Although RT-PCR testing for SARS-CoV-2, the mainstay of COVID-19 diagnosis, was widely available in many developed countries, its availability was extremely limited in Japan at the beginning of 2020 because Japanese central government policies restricted the provision of RT-PCR testing for SARS-CoV-2 and alternatively sealed off facilities where cluster infections emerged as a proactive epidemiological investigation [7,8]. Based on these restricted guidelines provided by the Japanese central government, local public health officers often rejected the clinicians’ requirement for RT-PCR testing for SARS-CoV-2 [7].

Additionally, although personal protective equipment (PPE) was required for medical personnel caring for all patients with possible or confirmed COVID-19 during that period [9], a shortage of PPE burdened the medical system, including non-specialized hospitals dealing with patients affected by COVID-19 [10].

Lack of accessibility to RT-PCR tests, a small number of negative pressure rooms, and a constant short supply of PPE restricted the appropriate management of patients referred from other hospitals and patients admitted from the ED with fever or pneumonia. Patients with a definitive diagnosis of COVID-19 were admitted to dedicated wards; however, the handling of "suspicious" patients remained undecided.

When the number of RT-PCR tests was limited, we created a novel risk-scoring strategy, the Fukuchi-Manabe score, for infection control measures of patients with suspected COVID-19 to prevent nosocomial infections, promote efficient use of the limited number of negative-pressure rooms, and reduce excessive PPE consumption. This study is the first to report on the development of this scoring strategy. We conducted an observational prospective cohort study to develop and validate our newly created Fukuchi-Manabe score for the quarantine of patients with suspected COVID-19. Simultaneously, we evaluated nosocomial COVID-19 infections among healthcare providers, as described in another study [11].

## Materials and methods

This single-center observational prospective cohort study was performed to create and validate a novel COVID-19 risk-scoring strategy, the Fukuchi-Manabe score. We enrolled all adult participants aged ≥18 years referred from other hospitals for fever or pneumonia and admitted to the ED at Saitama Medical Center, Jichi Medical University, Japan, during the first and second waves of the COVID-19 pandemic, between March 1, 2020, and August 31, 2020. Our institution was a university teaching hospital with a tertiary emergency medical service located in Saitama City, bordering the northern side of Tokyo Metropolitan, Japan, with 628 beds. There were six negative-pressure rooms, and RT-PCR testing was outsourced to Saitama Health Center and a commercial clinical laboratory company. Participants were screened using the Fukuchi-Manabe score. The inclusion criteria were as follows: age ≥18 years and the need for hospitalization. Outpatients and individuals <18 years of age were excluded. All participants underwent SARS-CoV-2 RT-PCR testing of the nasal cavity immediately after scoring. A total of 224 participants were enrolled. All laboratory and imaging data for this study were obtained from patient medical charts. The data were collected between June 1, 2020, and August 31, 2020, and were assessed on September 10, 2020.

G*Power version 3.1.9.2 (Heinrich Heine University Düsseldorf, Düsseldorf, Germany) was utilized to determine the requisite sample size, employing specific parameters for the estimation. The test family was designated as "z tests," with the statistical test configured as "logistic regression input effect size as odds ratio." The chosen type of power analysis was "a priori: compute required sample size given α, power, and effect size," with two tails considered. Odds ratios of "4.75" were calculated, with Pr (Y=1∣X=1) H1 = "0.20" representing the alternative hypothesis (H1) denoting the estimated probability of COVID-19 occurrence as the Fukuchi-Manabe score increases, and Pr (Y=1∣X=1) H0 = "0.05" representing the null hypothesis (H0) indicating the estimated probability of COVID-19 occurrence as the Fukuchi-Manabe score decreases. The α error probability was set at "0.05," while the power (1-β error probability) was established at "0.8." Additionally, R2 other X was determined as "0," signifying that the Fukuchi-Manabe score stands as the sole predictor. The distribution of X was defined as "binominal," with X parm pi at "0.5" and X parm sigma at "1" [12,13]. The sample size was calculated to be 161. We expanded the actual data size to 224 to achieve a higher power.

Primary and secondary outcomes

The primary outcome was a positive SARS-CoV-2 RT-PCR result, and the secondary outcome was the occurrence of nosocomial infections during the study period.

Fukuchi-Manabe score

By reviewing articles on COVID-19 published up to the start of the study period, the history, symptoms, chest CT findings, and laboratory data that were relevantly suggestive or informative of COVID-19 were scored, totaled, and divided into four categories ("probable," “possible,” “less likely,” and “non-suspicious”) based on the likelihood of COVID-19. The scoring table consisted of three major categories (Table 1).

**Table 1:** Fukuchi-Manabe score COVID-19: coronavirus disease 2019, CT: computed tomography, BNP: brain natriuretic peptide, *: urine antigens, urine antigens of pneumococcus or *Legionella, *GGO: grand glass opacity, CHF: congestive heart failure, WBC: white blood cell, LDH: lactate dehydrogenase Reference range: lymphocyte: 1500-4000, WBC: 3900-9000, procalcitonin level: <0.5 ng/ml, LDH: 110-220 mU/ml, BNP: <18.4 pg/ml

Items		Point									
		4	3		2	1	0	-1	-2	-3	-4
Sick contact or travel history		Yes									
Taste or smell disorder						Yes					
Shaking chill									Yes		
Unresolving pneumonia					Yes						
Acute coronary syndrome								Yes			
CT findings	Lung field		Bilateral GGO dominant in the lower lobe and subpleural area		Bilateral GGO	Unilateral GGO					
	Pleural effusion/mediastinal lymph nodes swelling							Positive			
Lymphocyte (/μL)					Below 1000	1000-1500					
WBC						Below normal range					
Procalcitonin level						Negative		Positive			
LDH (U/L)						Elevated		Normal range			
BNP >200 (pg/mL) or CHF								Yes			
Blood culture											Positive
*Urine antigens									Positive		

**Figure 1:**
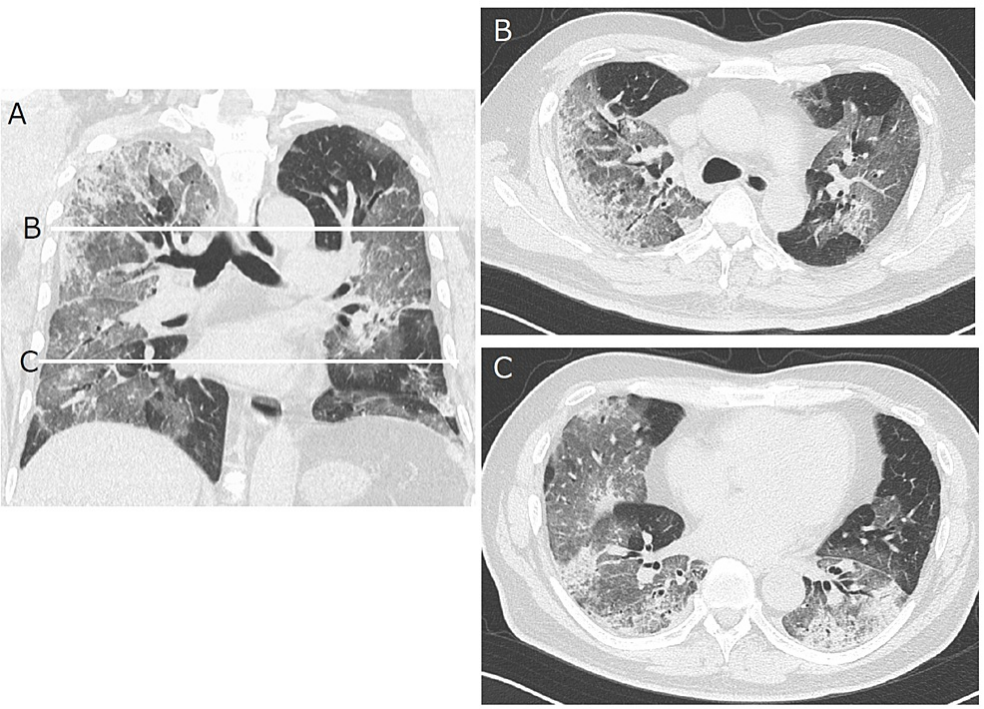
Chest CT imaging of typical COVID-19 pneumonia. Bilateral GGO dominant in the lower lobe and subpleural area makes a score of "3" using the Fukuchi-Manabe score (A) Coronal chest CT images (B). Just above the tracheal bifurcation level. (C) Heart level CT: computed tomography, COVID-19: coronavirus disease 2019, GGO: ground-glass opacity

**Figure 2:**
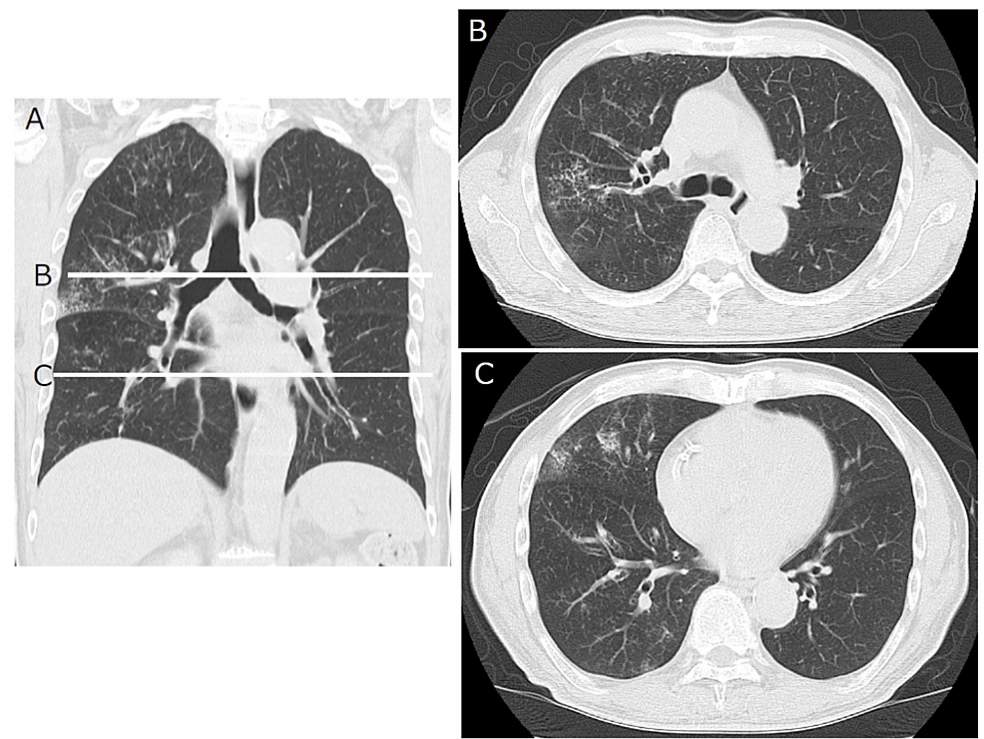
Chest CT imaging of atypical COVID-19 pneumonia. Unilateral GGO makes a score of "1" using the Fukuchi-Manabe score (A) Coronal chest CT images. (B) Tracheal bifurcation level. (C) Heart level CT: computed tomography, COVID-19: coronavirus disease 2019, GGO: ground-glass opacity

The first category included present illness, symptoms, and epidemiologic background, such as non-resolving pneumonia, evidence of shaking or chills, taste or smell disorder, contact with patients having COVID-19, and travel history [14,15]. The second category included chest CT findings, such as the distribution of ground-glass opacity (GGO) (Figure 1), absence of enlargement of mediastinal lymph nodes, pleural effusion, and signs of congestive heart failure (CHF) (Figure 2) [16]. The third category consisted of laboratory findings, such as lymphocyte count, white blood cell (WBC) count, procalcitonin level, lactate dehydrogenase (LDH) level, blood culture, urine antigens of pneumococcus or *Leginonella*, and brain natriuretic peptide (BNP) level [17,18]. Considering the first category, these parameters were chosen for their clinical simplicity and usefulness after the clinical course of COVID-19 was published in major articles. In particular, taste and olfactory dysfunction were applied as they were easily apparent to patients, even those without disease-specific findings. Although we understood that the patients’ declarations could be arbitrary, we still applied these parameters owing to their potentially high sensitivity.

Table 1 shows the electronic medical record chart introduced at our hospital, which was easily accessible to ED staff and medical residents. Depending on the total score, participants were classified into one of four categories: “probable,” over 4 points; “possible,” from 2 to 3 points; “less likely,” from 0 to 1 point; and “non-suspicious,” below 0 points. The admission ward, PPE level, and laboratory provisions for RT-PCR testing differed with the scores (Table 2). The cutoff values for the scores were arbitrary based on the choices of parameters. Because we were unable to perform the score creation and validation at different periods, we performed them simultaneously.

**Table 2:** Score stratification of the Fukuchi-Manabe score depicting differentiation of wards, PPE, and examination sites PPE: personal protective equipment, COVID-19: coronavirus disease 2019

Likelihood of COVID-19	Points	Ward	PPE	Examination submission site
Probable	≧4	Negative-air-pressure private room	Full PPE	Public health center
Possible	2-3	Negative-pressure private room or private room in normal wards		Commercial laboratory company
Less likely	0-1	Private room in a normal ward	Reduced PPE surgical mask, goggles, gloves	Consider a commercial laboratory company
Non-suspicious	≦-1	Normal	Normal	No need

Ethical approval

The study protocol was approved by the Institutional Clinical Research Ethics Review Board of Saitama Medical Center, Jichi Medical University, Japan (clinical #S21-036). It was performed following the tenets of the 1964 Declaration of Helsinki and its later amendments (revised in Tokyo in 2004). The requirement for written informed consent was waived, as an opt-out approach was used due to the observational nature of the study. Moreover, non-interventional methods were adopted, and only medical record information was utilized.

Statistical analyses

We evaluated the number of patients who were assessed using the Fukuchi-Manabe score and were positive on the RT-PCR test and the probability group to which they were assigned during the study period. The differences between the groups of RT-PCR positive for SARS-CoV-2 and RT-PCR negative for SARS-CoV-2 were tested using the Fisher's exact test for nominal variables and the Mann-Whitney U test for numerical variables.

A logistic regression model was used to establish the Fukuchi-Manabe score to predict positive RT-PCR test results. Receiver operating characteristic (ROC) analysis was performed to determine the sensitivity and specificity of scoring. The area under the curve (AUC) was validated using the bootstrap method (1,000 sets). The positive and negative predictive values of the “probable” category were evaluated.

Statistical software

The statistical package JMP Pro 16 (SAS Institute Ltd., Cary, NC, USA) was used for data analysis. G*Power version 3.1.9.2 (Heinrich Heine University Düsseldorf, Düsseldorf, Germany) was used for sample size calculation. Statistical significance was set at p<0.05.

## Results

Baseline information of patients

**Figure 3:**
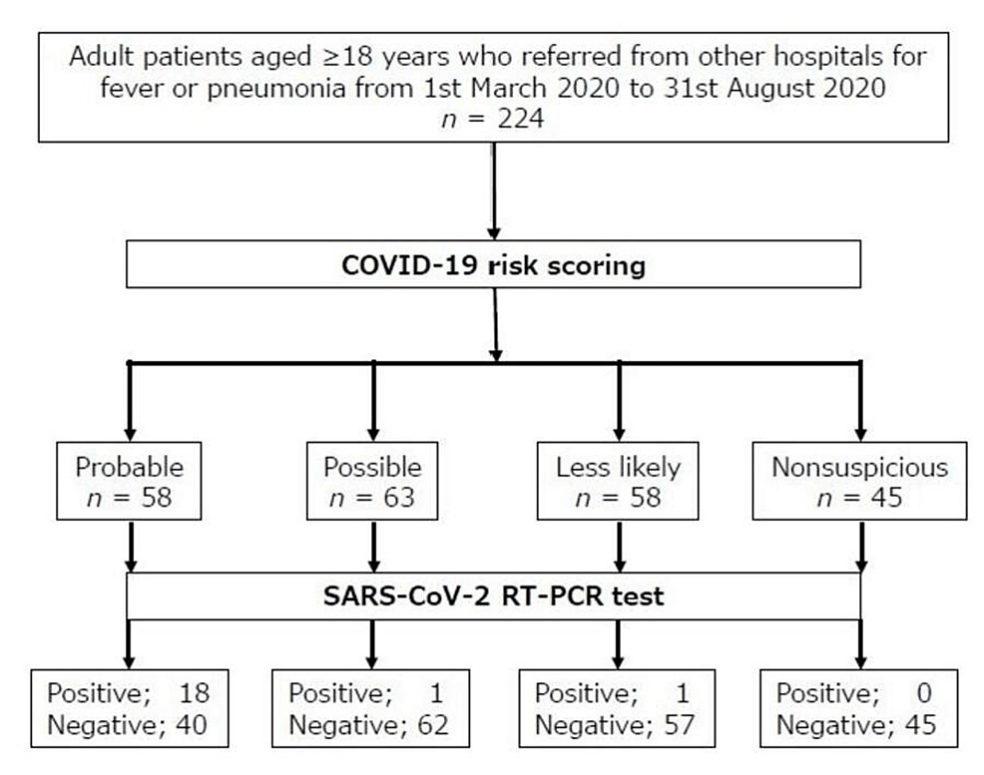
Patient flow in a prospective cohort study as per the Fukuchi-Manabe score COVID-19: coronavirus disease 2019, RT-PCR: reverse transcription-polymerase chain reaction, SARS-CoV-2: severe acute respiratory syndrome coronavirus 2

A total of 224 participants aged ≥18 years who were referred from other hospitals for fever or pneumonia and admitted to the ED underwent the Fukuchi-Manabe score and SARS-CoV-2 RT-PCR testing during the study period (Figure 3). The patient characteristics are summarized in Table 3. The distribution of the Fukuchi-Manabe score for each item of participants is summarized in Table 4. Of the 224 participants (157 male and 67 female), 20 (8.93%, 15 male and 5 female) were positive on the SARS-CoV-2 RT-PCR test, and these participants were significantly younger than the ones who received negative test results (p<0.000). WBC and BNP values were significantly lower in the SARS-CoV-2 RT-PCR-positive group than those in the negative group. The average scores of chest CT findings, contact with sick people, CHF, and total points were significantly higher in the SARS-CoV-2 RT-PCR-positive group than those in the negative group.

**Table 3:** Demographics of the participant n: participants, RT-PCR: reverse transcription-polymerase chain reaction, SARS-CoV-2: severe acute respiratory syndrome coronavirus 2, Me (2.5~97.5%): median value (2.5th and 97.5th percentiles), WBC: white blood cell, LDH: lactate dehydrogenase, BNP: brain natriuretic peptide, *: Mann-Whitney U test

Variables	n (%)	RT-PCR positive for SARS-CoV-2 Me (2.5~97.5%) {n}	RT-PCR negative for SARS-CoV-2 Me (2.5~97.5%) {n}	p-value^*^
Age (years)	224 (100)	54.5 (21.0~77.0) {20}	75.5 (24.0~93.4) {204}	0.000
Male, proportion (%)	157 (70.0)	75.0 {15}	69.6 {142}	0.615
WBC (/μL)	224 (100)	6325.0 (3610.0~20840.0) {20}	9575.0 (1366.0~26164.0) {142}	0.000
Lymphocyte count (/μL)	195 (87.1), missing 29	951.0 (192.0~2842.0) {17}	903.0 (161.0~4399.9) {178}	0.714
LDH (U/L)	224 (100)	320.5 (135.0~1463.0) {20}	283.5 (152.8~1261.8) {204}	0.599
BNP (pg/mL)	164 (73.2), missing 60	11.6 (4.0~178.2) {10}	154.7 (9.0~2870.5) {154}	0.000

**Table 4:** Distribution of the Fukuchi-Manabe score for each item by number of participants n: participants, RT-PCR: reverse transcription-polymerase chain reaction, SARS-CoV-2: severe acute respiratory syndrome coronavirus 2, WBC: white blood cell, LDH: lactate dehydrogenase, PCT: procalcitonin, CT: computed tomography, CHF: congestive heart failure, BNP: brain natriuretic peptide, Me (2.5~97.5%): median value (2.5th and 97.5th percentiles), #: Fisher's exact test

Item	Fukuchi-Manabe score	RT-PCR positive for SARS-CoV-2 n (%)	RT-PCR negative for SARS-CoV-2 n (%)	p-value^#^
WBC score, n (%)	1	1 (5.0)	15 (7.4)	1.000
	0	19 (95.0)	189 (92.6)	
Lymphocyte score, n (%)	0	6 (30.0)	37 (18.1)	0.480
	1	2 (10.0)	40 (19.6)	
	2	9 (45.0)	101 (49.5)	
	Missing	3 (15.0)	26 (12.7)	
LDH score, n (%)	-1	5 (25.0)	61 (29.9)	0.646
	1	15 (75.0)	143 (70.1)	
PCT score, n (%)	-1	5 (25.0)	66 (32.4)	0.294
	1	14 (70.0)	105 (51.5)	
	Missing	1 (5.0)	33 (16.2)	
CT score 1, n (%)	-1	0 (0.0)	21 (10.3)	0.000
	0	4 (20.0)	72 (35.3)	
	1	1 (5.0)	43 (21.1)	
	2	4 (20.0)	61 (29.9)	
	3	9 (45.0)	4 (2.0)	
	Missing	2 (10.0)	3 (1.5)	
CT score 2, n (%)	-1	1 (5.0)	67 (32.8)	0.010
	0	19 (95.0)	137 (67.2)	
Close contact, n (%)	0	9 (45.0)	200 (98.0)	0.000
	4	11 (55.0)	4 (2.0)	
Blood culture, n (%)	-4	0 (0.0)	24 (11.8)	0.599
	0	8 (40.0)	156 (76.5)	
	Missing	12 (60.0)	24 (11.8)	
Urinary antigen, n (%)	2	0 (0.0)	0 (0.0)	N/A
	0	5 (25.0)	89 (43.6)	
	Missing	15 (75.0)	115 (56.4)	
Decreased sense of taste or smell, n (%)	0	19 (95.0)	199 (97.5)	0.433
	1	1 (5.0)	5 (2.5)	
Chills or shivering, n (%)	-2	0 (0.0)	18 (8.8)	0.381
	0	20 (100.0)	186 (91.2)	
Refractory pneumonia, n (%)	0	18 (90.0)	197 (96.6)	0.187
	2	2 (10.0)	7 (3.4)	
Acute coronary syndrome, n (%)	-1	0 (0.0)	17 (8.3)	0.375
	0	20 (100.0)	187 (91.7)	
CHF or BNP >200, n (%)	-1	0 (0.0)	78 (38.2)	0.001
	0	20 (100.0)	126 (61.8)	
Total Fukuchi-Manabe score	Me (2.5~97.5%) {n}	6.0 (1.0~13.0) {20}	1.5 (-4.0~6.0) {204}	0.000

Results of primary and secondary outcomes

**Figure 4:**
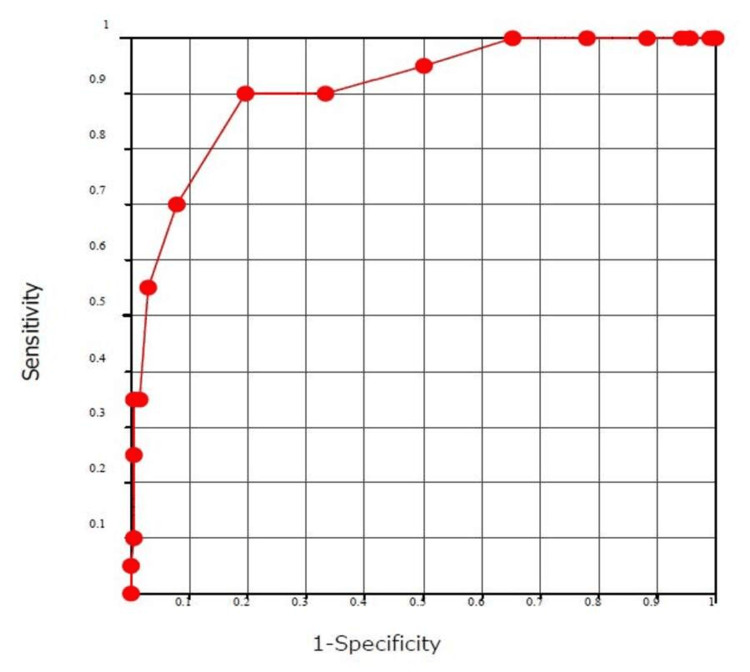
ROC curve of the Fukuchi–Manabe score. The AUC was 0.909 (95% confidence interval: 0.841–0.977), sensitivity was 90.0%, and specificity was 80.4% ROC: receiver operating characteristic, AUC: area under the curve

The score distribution was in the range of -7 to 13 (Table 5). True positives were evaluated from PCR-positive cases, with the most positive results (18 cases) obtained from the “probable” category (90.0%), while the remaining two positive results were obtained from “possible” or “less likely” cases. The AUC was 0.909 (95% confidence interval: 0.841-0.977), sensitivity was 90.0%, and specificity was 80.4%. The positive and negative predictive values and accuracy for the “probable” category were 0.90, 0.80, and 0.82, respectively. The AUC was validated using the bootstrap method (1,000 sets), with a mean of 0.910±0.034 (Figure. 4). No nosocomial infections among healthcare providers were observed during the study period, as previously reported.

**Table 5:** Distribution of the Fukuchi-Manabe score in the range from -7 to 13, depicting the likelihood of COVID-19 as probable, possible, less likely, and non-suspicious n: participants, RT-PCR: reverse transcription-polymerase chain reaction, SARS-CoV-2: severe acute respiratory syndrome coronavirus 2

Total Fukuchi-Manabe score	Fukuchi-Manabe score category	RT-PCR positive for SARS-CoV-2 n	RT-PCR negative for SARS-CoV-2 n	Total n
13	Probable	1	0	1
12		0	0	0
11		0	0	0
10		1	1	2
9		3	0	3
8		2	0	2
7		0	2	2
6		4	3	7
5		3	10	13
4		4	24	28
Subtotal for the category of “probable”		18	40	58
3	Possible	0	28	28
2		1	34	35
1	Less likely	1	31	32
0		0	26	26
-1	Non-suspicious	0	21	21
-2		0	12	12
-3		0	3	3
-4		0	7	7
-5		0	0	0
-6		0	1	1
-7		0	1	1
Subtotal for the categories of “possible,” “less likely,” and “non-suspicious”		2	164	166
Total for all four categories		20	204	224

Regarding the two outliers, the first participant had a score of 3 and was an older individual with dementia, fever, and myalgia. The visiting physician had tentatively diagnosed the participant with polymyalgia rheumatica and prescribed prednisolone. However, this participant was initially assessed as having low suspicion in the ED because of miscalculations and missing values and was admitted to a shared room. When he attended our department the next morning, the recalculated score placed him in the “possible” COVID-19 category. Consequently, the participant was immediately transferred to a private room. The SARS-CoV-2 RT-PCR results obtained later were positive; however, no nosocomial infections were observed.

The other outlier, a positive case with a score of 2, involved a man in his 50s who worked at a hotel and received RT-PCR results from a public health center. He denied obvious exposure to SARS-CoV-2; however, working at a hotel banquet might have led to unexpected exposure to the virus. He was referred to our hospital for respiratory distress on day 6. He had a history of mild chronic allergic rhinitis, which is why his score might be underestimated. Although the laboratory results were less positive than those of the other patients, CT imaging revealed typical unilateral COVID-19 pneumonia. The patient received only symptomatic treatment and was discharged after 13 days.

## Discussion

At the outset of the COVID-19 pandemic, the limited availability of RT-PCR testing and the shortage of PPE put a strain on healthcare systems worldwide, particularly in Japan. The Fukuchi-Manabe score strategy, which incorporated the relevant present illness history, chest CT findings, and laboratory data, was highly effective in stratifying patients with suspected COVID-19 into four different probability categories that helped guide appropriate ward and PPE management. Importantly, no nosocomial infections occurred during the study period. Although we were compelled to provide medical care at the beginning of the pandemic amid anxiety, confusion, fear, and stigma, clear orders and definitions facilitated the reduction of the burden on the hospital staff.

We aimed to establish the Fukuchi-Manabe score strategy with a high sensitivity and low specificity for infection control because false-negative results are much more problematic than false-positive results. The values for each item have been greatly simplified for the ease of understanding by the residents and nurses in the ED. The degree of suspicion was stratified into four levels and balanced to increase the sensitivity to ensure an adequate safety margin. The main focus of this study was early identification and screening. Each parameter was determined based on whether other diagnoses were likely and whether COVID-19-specific symptoms or findings were present. Although it would have been ideal to use multivariate analysis based on big data for scoring, in this case, the priority was the capability to score quickly in the ED; therefore, the numbers were set to ensure ease of scoring. When an outbreak of a new infectious disease occurs, clinicians are able to obtain the nearly complete picture because doctors and researchers worldwide quickly publish their studies and data.

Based on the medical history and physical examination, we emphasized the importance of close contact and travel history, as the disease is transmitted through droplets [19]. Olfactory and gustatory dysfunction are associated with COVID-19; moreover, it has been hypothesized that SARS-CoV-2 damages the olfactory pathways reaching the brain through the olfactory nerves, and these were considered positive findings [20]. Chills and shivering were findings strongly suggestive of sepsis or bacteremia [15]; therefore, a negative point was given if these were present. Certain antibiotics were administered as a preventive or treatment option for COVID-19; these were later withdrawn [21], and unresolved (post-antibiotic) pneumonia was still possible. Because CHF may present with shadows without an inflammatory response, we attributed a negative score for findings of obvious CHF.

Lung lesions, likely a result of COVID-19, received a high score, while lesions that were not as likely received a low score [22]. Although the CT results were almost always analyzed promptly by a radiologist, whenever radiologists were available, the scores were set based on the subjective judgment of the clinicians as 1-3 points according to the distribution of GGOs, with a higher point indicating peripheral or lower lesions. Pleural effusions and enlarged mediastinal lymph nodes were considered negative findings as they reduced the likelihood of COVID-19. Moreover, criticisms exist regarding subjecting patients to increased CT radiation exposure. There were concerns about the dose of radiation necessary. Nevertheless, early case reports of less symptomatic PCR-positive patients who already showed a GGO on CT showed that not performing CT imaging could be dangerous for patients.

The guidelines for COVID-19 diagnosis have not recommended CT screening [23]. Careful consideration should be given to the indications for CT, particularly in children, because high radiation exposure using CT is associated with a significantly increased rate of malignancy later in life [24]. However, as several CT scanners are available in Japan and can be used quickly in ED, including ours, it is a great advantage for diagnosis, especially during the period when the availability of RT-PCR testing was poor [24].

Several blood test findings were considered moderately specific for COVID-19, such as decreased WBC count, decreased lymphocytes, increased LDH levels, and positive procalcitonin levels [17,25], some of which are also apparent markers of severe diseases [26]. Although some case series have revealed that blood culture-positive complications might occur late in the clinical courses [27], a large negative score of -4 was given because viremia did not cause a positive blood culture. A positive urine antigen for pneumococcus or *Legionella* would reduce the possibility of COVID-19, although another diagnosis or colonization might coexist. As with physical examination and history, elevated BNP levels indicated a negative score.

After the scoring, each case was reassigned to a different room, PPE, and test submission institution. This was caused by limited negative-pressure rooms and private rooms, a shortage of PPE, and a limited number of RT-PCR tests. The “probable” group had a high probability of COVID-19 and was assigned to one of the few negative-pressure rooms. In contrast, the “possible” group with a slightly lower score was assigned normal private rooms without negative pressure. In both cases, full PPE was used. An advantage of this scoring strategy is that we did not observe any infection of medical personnel during the study period, even though, at the time, many healthcare workers at other institutions were infected or died as a result of COVID-19 [28]. No nosocomial infection among healthcare providers was observed at our institution. In contrast, nosocomial infections were frequently observed during the same period in Japan [29]. We used not only this scoring method but also other infection control measures, such as education for staff and self-quarantine. These measures facilitated the handling of complications by the medical staff without major disruptions.

During the initial period of the pandemic, an infectious disease specialist provided consultations for all suspected patients, who then decided on the clinical ward placement and correspondence depending on the medical records and images of the patients. The rapidly growing number of patients made it difficult to maintain this method; therefore, this scoring strategy, available to all medical staff at our hospital, reduced the burden on the infectious disease physician.

Limitations

This study had some limitations. First, the symptom-based nature of the scoring strategy makes it unsuitable for asymptomatic patients. Therefore, it cannot be used as a mass screening method to identify asymptomatic patients with COVID-19. Second, at the beginning of the global COVID-19 pandemic, the Japanese government and health authorities seemed to have deliberately avoided increasing the availability of RT-PCR tests, unlike in many other developed countries. For this reason, we could not perform sufficient RT-PCR tests, even though we were in a university hospital, and this problem was unique to Japan. Moreover, the restricted number of RT-PCR tests hindered the construction of a control group. It is worthy of note that the Japanese government later implemented policies to expand the availability of RT-PCR testing. Third, our hospital has few single-patient and negative-pressure rooms and no beds designated for patients with infectious diseases, a common situation in Japan. Fourth, the geographical situation of Saitama Prefecture was a limiting factor, as it has the lowest number of doctors per capita in Japan [7], despite being adjacent to the Tokyo metropolitan area and having many potential patients. Fifth, COVID-19 is a protean disease with mutating potential, leading to varied virulence and symptoms. In addition, introducing vaccines ameliorates disease severity; therefore, our findings on the wild type cannot be directly extrapolated to other variants. Finally, as this study was performed in the early period of the pandemic, this article was written as per the information available during that time.

Even with these limitations, the method described in this study will provide a basis for the adoption of similar procedures in the outbreaks of other novel pathogens [30] that constitute threats to humans. Until higher-modality tests, such as nucleic acid amplification tests, can be established for individual testing, a combination of clinical signs and other low-modality tests can temporarily protect strained healthcare systems.

## Conclusions

The Fukuchi-Manabe score, using relevant history of the present illness, chest CT findings, and selected laboratory data, facilitated the diagnosis and quarantine of patients with suspected COVID-19 during limited access to RT-PCR testing. This strategy minimizes nosocomial infections. Although specific examinations for COVID-19 have already been established, various measures were adopted in the process of developing such examinations. A risk-scoring strategy will be helpful to increase the likelihood of new diseases when another novel pathogen emerges in the future in the absence of or with limited specific genetic testing methods.
